# Pharmacokinetic Data Show That Oxolinic Acid and Flumequine Are Absorbed and Excreted Rapidly From Plasma and Tissues of Lumpfish

**DOI:** 10.3389/fvets.2019.00394

**Published:** 2019-11-12

**Authors:** Gyri T. Haugland, Karen O. Kverme, Rita Hannisdal, Marielle Kallekleiv, Duncan J. Colquhoun, Bjørn Tore Lunestad, Heidrun I. Wergeland, Ole B. Samuelsen

**Affiliations:** ^1^Department of Biological Sciences, University of Bergen, Bergen, Norway; ^2^Fish Health Research Group, Institute of Marine Research, Bergen, Norway; ^3^Fish Disease Group, Norwegian Veterinary Institute, Oslo, Norway

**Keywords:** pharmacokinetics, lumpfish, lumpsucker, oxolinic acid, flumequine, MIC, quinolones

## Abstract

This study examined the uptake, tissue distribution and elimination of the antibacterial agents oxolinic acid and flumequine in lumpfish (*Cyclopterus lumpus* L.) by use of LC-MS/MS following a single oral administration of 25 mg/kg fish given in feed. Lumpfish are increasingly used as cleaner fish for removal of sea lice on commercially farmed salmon. The production of lumpfish is successful, but there are challenges with bacterial infections and the number of antibacterial treatments has increased in recent years. As the lumpfish is a novel species to farming, there is a need for pharmacokinetic data and establishment of protocols for efficient antibacterial treatment. The current study describes the pharmacokinetic properties of oxolinic acid and flumequine in lumpfish. Absorption of oxolinic acid was moderate and was characterized by a calculated peak plasma concentration (C_max_) of 2.12 μg/ml after 10.3 h (T_max_) and an elimination half-life (t_1/2_β) of 21 h. Area under curve (AUC) and AUC from 0 to 24 h (AUC_0−24h_) were calculated to be 60.9 and 34.0 h μg/ml, respectively. For flumequine, plasma C_max_ was found to be 2.77 μg/ml after 7.7 h (T_max_) with t_1/2_β of 22 h. The area under the curve (AUC) and AUC from 0 to 24 h (AUC_0−24_) were calculated as 104.3 and 50.3 h μg/ml, respectively. Corresponding C_max_ values in muscle, liver, and head-kidney for oxolinic acid were 4.01, 3.04, and, 4.68 μg/g, respectively and T_max_ of 11.1, 9.2, and 10.0 h, respectively. For flumequine, C_max_ values of 4.16, 4.01, and 7.48 μg/g were obtained in muscle, liver, and head kidney, respectively, with corresponding T_max_ values of 10.2, 10.3, and 6.0 h. Antimicrobial susceptibility values as determined by minimum inhibitory concentration (MIC) analyses against 28 isolates of *Aeromonas salmonicida* isolated from diseased lumpfish ranged from 0.06 to 15 μg/ml for oxolinic acid and 0.024 to 6.25 μg/ml for flumequine. Bimodal distributions in susceptibility to both oxolinic acid and flumequine were observed. The combination of pharmacokinetic properties and MIC data make possible calculation of efficient treatment doses, which are needed to improve the welfare of lumpfish and minimize development of antibiotic resistant bacteria.

## Introduction

In recent years, lumpfish (*Cyclopterus lumpus* L.) and different species of wrasse are deployed in salmon farms as cleaner fish to removed lice from Atlantic salmon (1–3). While most of the wrasses are wild caught, the supply of lumpfish is based strictly on farmed individuals and the demand has led to an increase in the production of lumpfish in Norway, from around 0.4 million in 2012 to 30 million in 2018 (http://www.fiskeridir.no). However, like other fishes, lumpfish are susceptible to bacterial infections including those caused by atypical *Aeromonas salmonicida, Pasteurella* sp., *Pseudomonas anguilliseptica, Vibrio anguillarum*, and *Vibrio ordalii* (4–7). All lumpfish are vaccinated, but while currently available commercially vaccines show promising results, lumpfish are vulnerable to infections prior to vaccination and immediately post-vaccination, before vaccines can award protection (8, 9). *Pasteurella* sp. and *P. anguilliseptica* are not yet included in the vaccines. Over the last 7 years, the frequency of identification of pathogenic bacteria in lumpfish has increased significantly, from 12 cases in 2012 to 61 cases in 2018 (5). To treat a bacterial infection, the use of antibacterial agents can be appropriate. In Norwegian salmon farming, the consumption of antibacterial agents has been low for the last 25 years reflecting the low impact of bacterial infections in this species (statistics provided by www.fhi.no). Still, there has been an increase in the number of registered prescriptions in recent years due to treatment of lumpfish. However, as lumpfish are generally very small at the time of treatment this contributes little to the total volume of antibacterial agents used (10).

Today, the two antibacterial agents commercially available as medicated feeds in Norway are oxolinic acid (OA) and florfenicol (FFC). FFC is a thiamphenicol derivative, while OA and flumequine (FLU) belong to the group of antibacterial agents called quinolones. Quinolones can be effectively administered orally in fish via medicated feed and possess excellent antibacterial activity against common bacterial infections caused by *A. salmonicida* subsp. *salmonicida* (furunculosis), atypical *A. salmonicida* (atypical furunculosis), *V. anguillarum* (classical vibriosis), *Vibrio salmonicida* (cold-water vibriosis), and *Yersinia ruckeri* (yersiniosis). Therefore, quinolones have been widely used to treat systemic bacterial infections in fish (11–18). The pharmacokinetics of OA and FLU have been studied in cold water species like Atlantic salmon, Atlantic cod (*Gadus morhus* L.) and Atlantic halibut (*Hippoglossus hippoglossus* L.) (19–24). These studies show that species dependant differences in pharmacokinetic parameters are common for both FLU and OA. Bioavailability values of 65 and 31%, and plasma elimination half-lives (t_1/2_ β) of 74 and 43 h have been reported for FLU in Atlantic cod and Atlantic halibut, respectively (20, 25). For OA, major differences in bioavailability were identified in Atlantic cod and Atlantic halibut with values of 55 and 15% reported, respectively (21, 24). The largest difference in elimination rate (t_1/2_β) for OA was between Atlantic cod and Atlantic salmon with 84 and 15 h, respectively. These differences clearly show that the pharmacokinetic properties of a drug should always be investigated in the species in which it is intended to be used.

A practical application of pharmacokinetic data is the design of treatment regimens and prediction of possible clinical outcomes. To establish a correct dosage regime and promote optimal use of an antibacterial agent, both the pharmacokinetic properties of the drug and the susceptibility of the pathogen to the compound are required.

The aim of this study was to examine the pharmacokinetic properties of the quinolones OA and FLU in lumpfish following oral administration, and to relate these data to the minimum inhibitory concentration (MIC) values for *A. salmonicida* strains isolated from diseased lumpfish.

## Materials and Methods

### Experimental Fish

Unvaccinated lumpfish (*C. lumpus* L.) were obtained from Fjord Forsk AS (Sogndal, Norway), transported to the Aquatic and Industrial Laboratory (ILAB), Bergen, Norway, and kept in flow-through storage tanks (500 l) until the fish reach a mean weight of 113.5 ± 25.0 g and a length of 11.7 ± 1 cm. The seawater had a salinity of 34‰, a temperature of 12.0 ± 0.5°C and a flow-rate of ~1,000 l/h. The fish were fed a non-medicated ration of 1% body weight per day of dry pellets (Amber Neptun, 1.5 mm pellets; Skretting, Norway) and were starved for 2 d prior to- and 24 h post- antimicrobial administration. The experiment was approved by the Norwegian Food Safety Authority (ID 10178).

### Administration of Feed

Commercially available OA medicated feed (5 g kg^−1^, Skretting) was utilized. FLU medicated feed (5 g kg^−1^) was made by coating 1.5 mm Amber Neptun pellets (Skretting, Norway) with a premix of (1:1 w/w) glucose:FLU (Sigma). The final in-feed concentration of FLU was determined to be 5.2 ± 0.8 g per kg using LC-MS/MS as described below. The pellets were mixed 1:1 with milliQ water and homogenized as described previously (26) to make a paste, which was easily administered to the fish via a silicone hose and a syringe. The amount of feed administered to each fish corresponded to a dose of 25 mg OA or 25 mg FLU per kg fish, which is the dose recommended from the feed producer. Permission to make medicated feed with FLU was obtained from The Norwegian Medicines Agency.

### Sampling

To ensure as accurate sampling as possible for the first four samplings, four groups of six fish were placed in individual tanks (15 l, with through flow) following p.o. administration. The remaining fish were kept in a 500 l tank. Samples were taken at 3, 6, 12, 24, 48, 96, 144, 192, and 240 h post-administration for OA, and at 3, 6, 12, 24, 48, 72, 96, 120, and 168 h post-administration for FLU. At every sampling point, six fish were killed by a blow to the head and samples of plasma, muscle, head kidney and liver tissues were obtained. Blood (0.2–0.5 ml) was sampled from the caudal vein using a 1 ml syringe. Plasma was isolated by centrifugation of blood at ~2,000 g for 10 min. All samples were immediately frozen and stored at −20°C until analyzed.

### Analyses of FLU and OA

For the analysis of FLU an internal standard (FLU-^13^C3; Sigma Aldrich) was added to the homogenized tissue samples and plasma (0.1 ml). The analytes were extracted using 0.5% formic acid in acetonitrile. The samples were vortex-mixed and centrifuged before the extracts were transferred to a new vial and concentrated at 40°C under nitrogen flow. The residues were dissolved in water/methanol (60:40) and filtered through a 0.45 μm filter. Analysis was performed using an Agilent 1290 LC-system (Agilent Technologies) coupled to an Agilent 6460 triple quadrupole mass spectrometer (Agilent Technologies). The instrument was equipped with an electrospray ionization ESI source operated in positive mode. The analytes were separated by a reverse phase Agilent stable bond C18-column (150 × 2.1 mm i.d., 1.8 μm particle size) (Agilent Technologies) using a 0.4 ml/min flow. The mobile phase was a mixture of methanol and 0.1% formic acid in water. Chromatography was performed utilizing a stepwise gradient: 0–0.3 min, 20% methanol; 1.5 min, 80% methanol; 2.1–2.1 min, 95% methanol; 2.2–4.0 min, 20% methanol. All gradient steps were linear. The retention time for FLU and FLU-^13^C3 was 2.0 min. The following source conditions were used: gas temperature: 200°C; gas flow: 6 l/min; nebulizer pressure: 35 psi; sheath gas temperature: 350°C; sheath gas flow: 12 l/min; capillary voltage: 4,000 V; nozzle voltage: 0 V. The analytes were monitored using the following transitions: 262.9 m/z → 245.0 m/z (quantifier) and 262.9 m/z → 203.0 m/z (qualifier); FLU-^13^C3, 265.9 m/z → 248.0 m/z. Procedural blank, matrix blank, matrix-matched calibration curve and controls were prepared for each series. The limit of quantification (LOQ) for FLU was determined as 10 ng/g in tissues, and 10 ng/ml in plasma. The method was linear over the range studied (LOQ-−25,000 ng/g). Recovery ranged from 90 to 110%, and relative standard deviation was <10%.

The analysis of OA was performed in a similar manner as described for FLU. However, OA-d5 (Sigma Aldrich) was used as internal standard and acetonitrile was used for extraction. The retention time for OA and OA-d5 was 1.8 min. OA and the internal standard were monitored using the following transitions: OA, 262.9 m/z → 245.0 m/z (quantifier) and 262.9 m/z → 217.0 m/z (qualifier); OA-d5, 267.9 m/z → 250.0 m/z. Procedural blank, matrix blank, matrix-matched calibration curve and controls were prepared for each series. The LOQ for OA was determined to 2.0 ng/g in tissue and 2.0 ng/ml in plasma. OA was linear up to 15 000 ng/g. Recovery ranged from 90 to 110%, and relative standard deviation was <10%.

### Pharmacokinetic Analysis

Standard pharmacokinetic parameters were calculated using the computer program PCNONLIN version 4.2 (Statistical Consultants Inc., Lexington, KY, USA) using the best-fit relationship between mean plasma or tissue drug concentrations and time. Best-fit models were chosen using Akaike's information criterion estimation in which all data were weighted to produce the best-fit curve (27). Elimination half-life (t_1/2_β) was calculated using logarithmically (ln) transformed drug concentrations in the elimination phase vs. time using the formula t_1/2_ = ln2/k, where k is the slope of the regression line.

### Bacterial Culture

Twenty-eight isolates of *A. salmonicida* isolated from diseased lumpfish from different locations in Norway were cultured in tryptic soy broth (TSB) at 20°C, 200 rpm until late log phase. The number of cells were determined using the cell counter CASY Modell TT 150 μm (Roche Diagnostics) and diluted to a concentration of 5 × 10^6^ bacteria ml^−1^.

### Minimum Inhibitory Concentration (MIC) Determinations

The MICs were performed using microtiter plate with 96-well with rounded bottom (Sarstedt AG & Co.). A 2-fold dilution of OA and FLU (Sigma) suspended in TSB in the range of 0.00002–60 μg/ml and 0.0008–400 μg/ml, respectively, were performed. Three parallels were performed for each concentration. One hundred microliters of bacterial suspension (5 x 10^6^ bacteria/ ml) were mixed with 100 μl of antibacterial agent diluted in TSB. Negative controls containing bacterial suspension, but no antibacterial agents were included for each isolate. The plates were sealed with microseal “B” PCR Plate sealing film (BIORAD) and incubated at 20°C for 48 h. The MICs were determined after visual inspections, and given as the concentrations where no growth was observed.

### Statistical Analyses

Analysis of variance (ANOVA) was performed using the statistical software package SigmaStat 3.5 to evaluate the effect of time. If the variance was not normally distributed, the *P*-value cut off was set to 0.01 as suggested by Glass et al. (28). The Holm-Sidak method was performed for pairwise multiple comparison.

## Results

**Figure 1:**
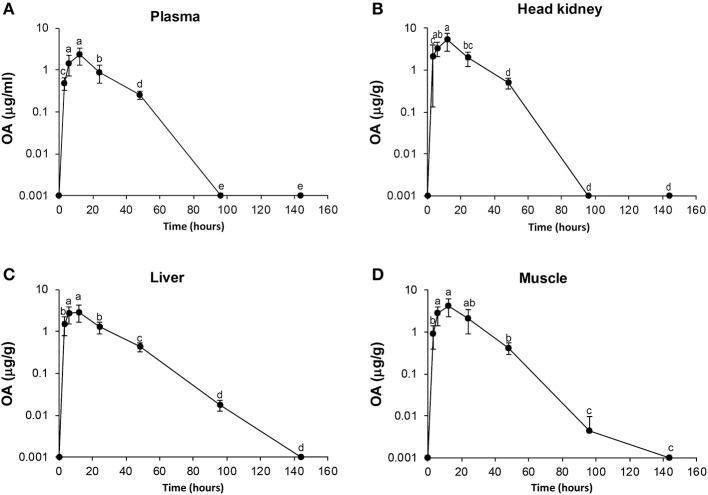
Diagrams of uptake and elimination of oxolinic acid (OA) at different time points post oral administration of medical feed (25 mg/kg). Concentrations of OA in plasma **(A)**, head kidney **(B)**, liver **(C)**, and muscle **(D)**. Time points are significant different statistically if they do not shear letter. Measurements above 160 h were lower than LOQ and not included in the Figure for better visualization.

The mean concentrations of OA and FLU in plasma, muscle, liver, and head-kidney obtained from a single oral administration to six fish at each sampling point are shown in Tables 1, 2, respectively. For plasma and all tissues, the highest level of OA was measured 12 h post-administration (Figure 1). The highest concentration at 12 h post-administration was in head kidney, followed by muscle, liver, and plasma. Statistically significant differences are shown in the Figure 1 as single letters. Plasma data were used to calculate the pharmacokinetic parameters using PCNONLIN. Plasma data for OA was best described by an open one-compartment model with first-order input, first-order output and a lag-time. The peak plasma concentration (C_max_) was calculated to be 2.12 μg/ml, the time to peak plasma concentration (T_max_) to be 10.3 h and the absorption (${\text{t}}_{½}\alpha$) and elimination halflife (${\text{t}}_{½}\beta$) to be 6 and 21 h, respectively. Area under the curve (AUC) and AUC from 0 to 24 h (AUC_0−24h_) were calculated to be 60.9 and 34.0 h μg/ml, respectively.

**Table 1:** Mean concentration of oxolinic acid (OA) in plasma (μg/ml) and tissue (μg/g) at different time points post oral administration of 25 mg/kg.

**Sample**	**Time (h)**									
	**0**	**3**	**6**	**12**	**24**	**48**	**96**	**144**	**192**	**240**
Plasma	LOQ	0.49 ± 0.17	1.47 ± 0.75	2.23 ± 1.01	0.87 ± 0.40	0.25 ± 0.06	LOQ	LOQ	LOQ	LOQ
Head kidney	LOQ	2.05 ± 1.92	3.32 ± 1.18	5.26 ± 2.39	1.97 ± 0.75	0.49 ± 0.15	LOQ	LOQ	LOQ	LOQ
Liver	LOQ	1.52 ± 0.73	2.67 ± 1.14	2.91 ± 1.28	1.27 ± 0.39	0.43 ± 0.10	0.02 ± 0.01	LOQ	LOQ	LOQ
Muscle	LOQ	0.90 ± 0.52	2.75 ± 1.31	4.25 ± 1.95	2.12 ± 1.22	0.42 ± 0.13	0	LOQ	LOQ	LOQ

**Table 2:** Mean concentration of flumequine (FLU) in plasma (μg/ml) and tissue (μg/g) at different time points post oral administration of 25 mg/kg.

**Sample**	**Time (h)**									
	**0**	**3**	**6**	**12**	**24**	**48**	**72**	**96**	**120**	**168**
Plasma	LOQ	1.91 ± 0.59	2.93 ± 0.87	2.50 ± 0.47	1.49 ± 0.65	0.34 ± 0.12	0.09 ± 0.13	LOQ	0.02 ± 0.04	LOQ
Head kidney	LOQ	6.23 ± 5.47	7.48 ± 3.20	4.88 ± 1.03	3.25 ± 1.89	0.45 ± 0.26	0.08 ± 0.12	LOQ	0.01 ± 0.02	LOQ
Liver	LOQ	4.14 ± 1.69	6.08 ± 2.79	4.69 ± 0.82	2.48 ± 1.03	0.66 ± 0.25	0.18 ± 0.25	0.03 ± 0.01	0.02 ± 0.04	LOQ
Muscle	LOQ	2.27 ± 0.88	3.49 ± 0.73	4.23 ± 1.02	2.32 ± 1.37	0.26 ± 0.19	0.032 ± 0.05	LOQ	0	LOQ

**Figure 2:**
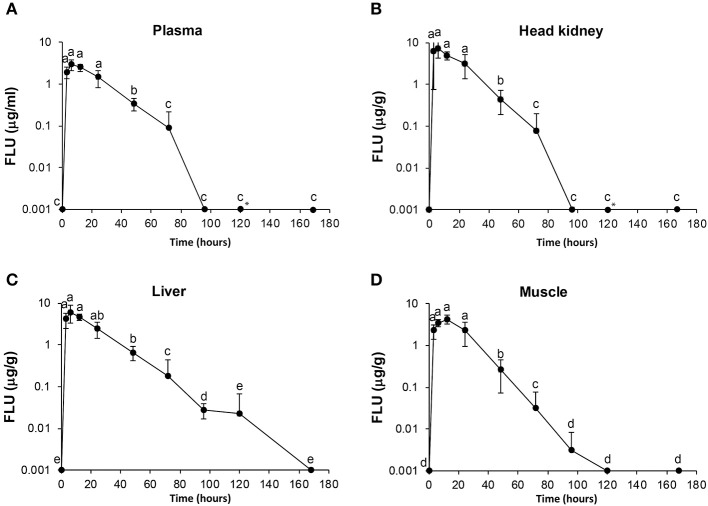
Diagrams of uptake and elimination of flumequine (FLU) at different time points post oral administration of medical feed (25 mg/kg). Concentrations of FLU in plasma **(A)**, head kidney **(B)**, liver **(C)**, and muscle **(D)**. Time points are significant different statistically if they do not shear letter. *Measurement in one of six fish at 120 h was an outlier and not included in the diagram.

For FLU, the highest concentration in plasma, head kidney and liver was measured 6 h post-administration, while for muscle, the highest concentration of FLU was 12 h post-administration. Statistical significant differences are shown in Figure 2 as single letters. Plasma data for FLU were best described by an open one-compartment model with first-order input, first-order output and no lag-time. Plasma C_max_, T_max_, ${\text{t}}_{½}\alpha$, ${\text{t}}_{½}\beta$, AUC, and AUC_0−24h_ for FLU were found to be 2.77 μg/ml, 7.7 h, 2.5 h, 22 h, 104.3, and 50.3 h μg/ml, respectively. The calculated pharmacokinetic values for OA and FLU in plasma, muscle, head kidney and liver are given in Tables 3, 4, respectively.

**Table 3:** Calculated pharmacokinetic parameters for oxolinic acid in plasma, muscle, head kidney, and liver of lumpfish following a single oral administration of 25 mg/kg.

**Tissue**	**AUC**	**AUC_0−24_**	**C_max_**	**T_max_ (h)**	**T_1/2_β (h)**
Plasma	61 h μg/ml	34 h μg/ml	2.1 μg/ml	10.3	21
Muscle	137 h μg/g		4.0 μg/g	11.1	15
Liver	102 h μg/g		3.0 μg/g	9.2	22
Head kidney	139 h μg/g		4.7 μg/g	10.0	19

*AUC, Area Under Curve; AUC_0−24_, Area Under Curve from 0 to 24 h; C_max_, maximum concentration; T_max_, time to maximum concentration; t_1/2_β, elimination half-life*.

**Table 4:** Calculated pharmacokinetic parameters for flumequine in plasma, muscle, head kidney (H-kidney), and liver of lumpfish following a single oral administration of 25 mg/kg.

**Tissue**	**AUC (h μg/ml)**	**AUC_**0−24**_ (h μg/ml)**	**C_**max**_**	**T_**max**_ (h)**	**T_1/2_β (h)**
Plasma	104 h μg/ml	50 h μg/ml	2.8 μg/ml	7.7	22
Muscle	144 h μg/g		4.2 μg/g	10.2	15
Liver	198 h μg/g		4.0 μg/g	10.3	22
Head kidney	217 h μg/g		7.5 μg/g	6.0	24

*AUC, Area Under Curve; AUC_0−24_, Area Under Curve from 0 to 24 h; C_max_, maximum concentration; T_max_, time to maximum concentration; t_1/2_β, elimination half-life. Data for head-kidney did not fit any model and C_max_ and T_max_ values are therefore from Table 2*.

Susceptibility (MIC) to OA and FLU was tested in 28 isolates, typical and atypical, of *A. salmonicida* isolated from diseased lumpfish. The distribution of MIC‘s for the atypical isolates (*n* = 22) was from 0.024 to 0.2 μg/ml with a median wild type (WT) MIC of 0.098 for FLU and 0.23 μg/ml for OA (0.059–0.47 μg/ml). One atypical isolate displayed a MIC value of 3.78 μg/ml for OA and 0.78 μg/ml for FLU. The typical *A. salmonicida* isolates (*n* = 5) had a distribution of MIC‘s from 3.75 to 15 μg/ml for OA and 0.78 to 6.25 μg/ml for FLU.

## Discussions

Studies have revealed major variances in the pharmacokinetic properties of OA and FLU in different fish species, supporting the argument that such studies should be performed in the species for which a particular compound is intended used. In the current study, we have used LC-MS/MS, which is an rapid method to determine the concentration of drug residues (29). In previous study, we used this method for determination of uptake and elimination of florfenicol in lumpfish (26).

Following a single oral administration of OA or FLU to lumpfish, a one-compartment open model with first-order input and first-order output best described the mean plasma concentrations vs. time curve for both compounds. This model was previously used to describe single oral administration of OA in Atlantic salmon, Atlantic halibut, and Atlantic cod (19, 21, 22, 24) and FLU in Atlantic salmon (19). In Atlantic halibut, however, single oral administration of FLU was best described by a two-compartment model (20).

T_max_ is a suitable indicator to describe the absorption rate following an oral administration of a drug. In lumpfish, the plasma T_max_ for OA was calculated to 10.3 h indicating faster absorption than in Atlantic halibut, Atlantic cod, Atlantic salmon, rainbow trout (*Oncorhynchus mykiss* Walbaum, 1792) and sharpsnout sea bream (*Diplodus puntazzo* Walbaum, 1792) where plasma T_max_ values ranged from 19 to 24 h (21, 22, 24, 30–32). While the absorption rate for FLU was fast in lumpfish (plasma T_max_ of 7.7 h) considerable variation in plasma T_max_ has been documented in other fish species, with 6, 10, and 13.7 h in Atlantic salmon, 7 h in turbot (*Scophthalmus maximus* L.), 7 and 20 h in Atlantic halibut and 24 h in Atlantic cod (19, 20, 25, 33, 34). In a recent study, a T_max_ of 21.2 h was found for FFC in lumpfish held at 12°C (26), hence a significantly slower absorption rate compared to FLU and OA.

The maximum plasma concentration (C_max_) of 2.12 μg/ml for OA in lumpfish was higher than in Atlantic cod and Atlantic halibut (1.2 μg/ml) (21), and Atlantic salmon (0.61 and 0.87 μg/ml), using the same dose (19, 24, 31). A single administration of 30 mg/kg gave C_max_ of 0.92 in sharpsnout sea bream (32). The plasma C_max_ for FLU was calculated to 2.77 μg/ml in lumpfish and is thus comparable to Atlantic halibut (2.7 μg/ml) and Atlantic salmon (2.6 μg/ml) (21, 34). In comparison, a single administration of 10 mg/kg gave C_max_ of 3.5 μg/ml in Atlantic cod and 1.9 μg/ml in turbot (24, 25).

Since single intravenous injection of the drugs was not included in this study, the distribution volume (Vd) could not be determined. However, an alternative method to describe the distribution of a compound from blood to tissues is to use the AUC values in Tables 3, 4 to estimate tissue/plasma ratios. For OA these ratios were 2.3, 1.6, and 2.3 for muscle, liver, and head kidney, respectively, in lumpfish. Corresponding ratios for FLU were 1.3, 1.9, and 2.1 respectively and indicate a satisfactory tissue distribution for both compounds. In muscle and liver of Atlantic salmon, the tissue/plasma ratios for OA where calculated to 5.7 and 5.9, respectively, demonstrating a much better distribution of OA in this species compared to lumpfish (22). In a similar pharmacokinetic study of FFC in lumpfish, only the head kidney/plasma ratio exceeded 1.0, indicating a substantial lower distribution of florfenicol (26).

Plasma elimination half-lives (t_1/2_β) of OA and FLU in fish are temperature and species dependent. An elimination half-life of 21 h in lumpfish is comparable to reported t_1/2_β values of 18.2 h (10.2°C) and 18 h (10°C) for OA in Atlantic salmon, and shorter than in Atlantic cod and halibut which demonstrated plasma t_1/2_β values of 82 and 48 h (8°C), respectively (19, 21, 22, 24). Sea bream eliminate OA at a high rate with calculated half-lives of 10 and 12.6 h at 19^o^C (28). A similar tendency was seen for FLU with t_1/2_β values of 22 h in lumpfish and 22.4 and 22.8 h in Atlantic salmon (19, 35) compared to 74 h in Atlantic cod and 43 h in Atlantic halibut at temperatures of 8 and 10°C, respectively (20, 33). The half-lives of OA and FLU in lumpfish range from 15 h in muscle to 22 h in liver for OA and from 15 in muscle to 24 h in head kidney for FLU. Hence, minor difference in elimination half-lives between the organs.

To maximize treatment efficacy and minimize the risk of development of bacterial resistance, pharmacokinetic knowledge combined with susceptibility tests (MIC), are important tools for establishment of optimal antibacterial dosage regimes. A peak concentration C_max_/MIC ratio of at least 8 has been suggested to obtain maximum efficacy and prevent resistance development for bactericidal drugs that act mainly by concentration-dependant mechanisms such as FLU and other fluoroquinolones (36). Although OA is listed as a bacteriostatic drug (Summary of Product Characteristics (SPC), www.felleskatalogen.no/medisin-vet.), Barnes et al. (37) suggested that the activity of OA against *A. salmonicida* was concentration dependent and Wright et al. (38) recommended that the mode of action of the quinolones is best considered as concentration dependent. We have therefore evaluated the efficacy of both OA and FLU using C_max_/MIC ratio as the PK/PD indices. The relevant pharmacokinetic parameter for OA and FLU is plasma C_max_ which is 2.12 and 2.77 μg/ml for these substances, respectively. In the literature describing the pharmacokinetics of OA and FLU in fish, the plasma concentrations of the drugs are given as total concentration, including free and bound to plasma protein. If the total drug concentration was used in setting C_max_/MIC ≥ 8 for the PK/PD indices, then the critical breakpoint MIC values (C_max_/8) could be estimated to 0.265 and 0.346 μg/ml for OA and FLU, respectively. This indicates clinical efficacy for most of the atypical strains using OA and all atypical strains using FLU while all typical strains tested can be classified as resistant for both drugs, indicating low or no clinical efficacy. If, on the other hand, free drug was used in setting ≥8 for C_max_/MIC, the C_max_ value must be adjusted accordingly. The plasma protein binding of OA and FLU in lumpfish is, as for many fish species, unknown. Bjørklund and Bylund (39) reported a 27% plasma protein binding of oxolinic acid in rainbow trout and Plakas et al. (40) found variable but saturable plasma protein binding (88–55% at 0.125–8.0 μg/ml) of FLU in channel catfish (*Ictalurus punctatus* Rafinesque, 1818). Assuming a plasma protein binding of 27 and 55%, respectively for OA and FLU in lumpfish, the C_max_ values of free drugs can be calculated to 1.54 μg/ml for OA and 1.25 μg/ml for FLU and thereby altering critical breakpoint MIC values to 0.193 μg/ml for OA and 0.156 μg/ml for FLU.

## Conclusion

The availability of pharmacokinetic data and MIC determination provide an important theoretical basis for calculation of efficient antibacterial treatment and for calculation of suitable withdrawal periods. Our results indicate that an oral administration of 25 mg/kg of OA or 25 mg/kg FLU will only be sufficient in treating lumpfish infected with sensitive *A. salmonicida* isolates. For the strains that are less sensitive to quinolones, an increase in dose will be required to improve efficacy. Otherwise, alternatives like florfenicol should be considered. Sensitivity testing prior to treatment of diseased lumpfish is crucial and recommended doses should be tested experimentally. Effective treatment is important for reducing the risk of development of antimicrobial resistant bacteria and for the welfare of lumpfish.

## Data Availability Statement

All datasets generated for this study are included in the article/Supplementary Material.

## Ethics Statement

The animal study was reviewed and approved by Norwegian Food Safety Authority and The Norwegian Medicines Agency.

## Author Contributions

KK, GH, RH, and MK performed the experiments. GH, OS, RH, and MK analyzed data and performed statistical analyses. OS, GH, and RH wrote the initial draft. DC provided the isolates included in the MIC-analysis. GH, OS, DC, HW, RH, KK, MK, and BL were involved in planning and discussion. All authors gave suggestions in the formation and revision of the manuscript.

### Conflict of Interest

The authors declare that the research was conducted in the absence of any commercial or financial relationships that could be construed as a potential conflict of interest.

## References

[1] Imsland AK Reynolds P Eliassen G Hangstad TA Foss A Vikingstad E The use of lumpfish (*Cyclopterus lumpus* L.) to control sea lice (*Lepeophtheirus salmonis* Krøyer) infestations in intensively farmed Atlantic salmon (*Salmo salar* L.). Aquaculture. (2014) 425–6:18–23. 10.1016/j.aquaculture.2013.12.033

[2] Powell A Treasurer JW Pooley CL Keay AJ Lloyd R Imsland AK Use of lumpfish for sea-lice control in salmon farming: challenges and opportunities. Rev Aquacult. (2018) 10:683–702. 10.1111/raq.12194

[3] Treasurer J An introduction to sea lice and the rise of cleaner fish. In: Treasurer J, editor. In Cleaner Fish Biology and Aquaculture Application. Sheffield: 5M Publishing Ltd (2018). p. 3–25.

[4] Alarcon M Thoen E Poppe TT Bornø G Mohammad SN Hansen H. Co-infection of *Nucleospora cyclopteri* (Microsporidia) and *Kudoa islandica* (Myxozoa) in farmed lumpfish, *Cyclopterus lumpus* L., in Norway: a case report. J Fish Dis. (2016) 39:411–8. 10.1111/jfd.12372 25865243

[5] Walde C Gulla S Hansen H Bysveen M Bornø G Health situation for cleaner fish (In Norwegian). In: Hjeltnes B Jensen B Bornø G Haukaas A Walde CS, editors. Fish Health Report. Norwegian Veterinary Institiute (2019).

[6] Scholz F Glosvik H Maros-López M Cleaner fish health. In: Treasurer JW, editor. Cleaner Fish Biology and Aquaculture Applications. Sheffield: 5M publications (2018). p. 221–57.

[7] Rimstad E Basic D Gulla S Hjeltnes B Mortensen S Risk assessment of fish health associated with the use of cleaner fish in aquaculture. VKM Report 2017:32. In: Report from the Norwegian Scientific Commitee for Food and Environment (VKM). Oslo: Norwegian Scientific Commitee for Food and Environment (VKM) (2017).

[8] Haugland GT Rønneseth A Wergeland HI Immunology and vaccinology of lumpfish and wrasse. In: Cleaner Fish Biology and Aquaculture Applications. Treasurer J, editor. Sheffield: 5M publishing (2018). p. 258–80.

[9] Rønneseth A Haugland GT Colquhoun DJ Brudal E Wergeland HI. Protection and antibody reactivity following vaccination of lumpfish (*Cyclopterus lumpus* L.) against atypical *Aeromonas salmonicida*. Fish Shellfish Immunol. (2017) 64:383–91. 10.1016/j.fsi.2017.03.040 28344167

[10] Grave K Helgesen KO Antibacterial Agents to Farmed Fish and Cleaner Fish – Requisition, Consumption and Diagnoses 2013-2017 (In Norwegian). Oslo: The Norwegian Veterinary Institute (2018).

[11] Austin B Rayment J Alderman DJ Control of furunculosis by oxolinic acid. Aquaculture. (1983) 31:101–8. 10.1016/0044-8486(83)90305-8

[12] Chevalier R Gerard JP Michel C Distribution et cinétique tissulaire de la flumequine chez la truite arc-en-ciel (*Salmo gairdneri*, Richardson). Rev Méd Vét. (1981) 132:831–7.

[13] Hustvedt SO Salte R Vassvik V Combatting cold-water vibriosis in Atlantic salmon (*Salmo salar* L.) with oxolinic acid: a case report. Aquaculture. (1992) 103:213–9. 10.1016/0044-8486(92)90167-J

[14] Rogers CJ Austin B Oxolinic acid for control of enteric redmouth disease in rainbow trout. Vet Rec. (1983) 112:83–6. 10.1136/vr.112.4.83 6829150

[15] Samuelsen OB Bergh Ø Efficacy of orally administered florfenicol and oxolinic acid in the treatment of vibriosis in cod (*Gadus morhua* L.). Aquaculture. (2004) 235:27–35. 10.1016/S0044-8486(03)00446-0

[16] Guidi LR Santos FA Ribeiro A Fernandes C Silva LHM Gloria MBA. Quinolones and tetracyclines in aquaculture fish by a simple and rapid LC-MS/MS method. Food Chem. (2018) 245:1232–8. 10.1016/j.foodchem.2017.11.094 29287347

[17] Samuelsen OB Kvenseth PG Andreassen JH Torkildsen L Ervik A Bergh O. The efficacy of a single intraperitoneal injection of oxolinic acid in the treatment of bacterial infections in goldsinny wrasse (*Ctenolabrus rupestris*) and corkwing wrasse (*Symphodus melops*) studied under field and laboratory conditions. J Vet Pharmacol Ther. (2003) 26:181–6. 10.1046/j.1365-2885.2003.00478.x 12755901

[18] Samuelsen OB Pharmacokinetics of quinolones in fish: a review. Aquaculture. (2006) 255:55–75. 10.1016/j.aquaculture.2005.12.008

[19] Martinsen B Horsberg TE. Comparative single-dose pharmacokinetics of four quinolones, oxolinic acid, flumequine, sarafloxacin, and enrofloxacin, in Atlantic salmon (*Salmo salar*) held in seawater at 10 degrees C. Antimicrob Agents Chemother. (1995) 39:1059–64. 10.1128/AAC.39.5.1059 7625789 PMC162683

[20] Samuelsen OB Ervik A Single dose pharmacokinetic study of flumequine after intravenous, intraperitoneal and oral administration to Atlantic halibut (*Hippoglossus hippoglossus*) held in seawater at 9C. Aquaculture. (1997) 158:215–27. 10.1016/S0044-8486(97)00190-7

[21] Samuelsen OB Ervik A A single-dose pharmacokinetic study of oxolinic acid and vetoquinol, an oxolinic acid ester, in Atlantic halibut (*Hippoglossus hippoglossus*) held in seawater at 9°C. J Fish Dis. (1999) 22:13–23. 10.1046/j.1365-2761.1999.00133.x

[22] Samuelsen OB Pursell L Ervik A Smith P A single-dose pharmacokinetic study of oxolinic acid and vetoquinol, an oxolinic acid ester, in Atlantic salmon (*Salmo salar*) held in seawater and *in vitro* antibacterial activity against *Aeromonas salmonicida*. Aquaculture. (2000) 187:213–24. 10.1016/S0044-8486(00)00315-X

[23] Ellingsen OF Midttun B Rogstad A Syvertsen C Samuelsen OB Dosage regime experiments with oxolinic acid and flumequine in Atlantic salmon (*Salmo salar*) held in seawater. Aquaculture. (2002) 136:19–34. 10.1016/S0044-8486(01)00804-3

[24] Samuelsen OB Bergh O Ervik A. A single-dose pharmacokinetic study of oxolinic acid and vetoquinol, an oxolinic acid ester, in cod, *Gadus morhua* L., held in sea water at 8 degrees C and *in vitro* antibacterial activity of oxolinic acid against *Vibrio anguillarum* strains isolated from diseased cod. J Fish Dis. (2003) 26:339–47. 10.1046/j.1365-2761.2003.00466.x 12899409

[25] Hansen MK Horsberg TE. Single-dose pharmacokinetics of flumequine in halibut (*Hippoglossus hippoglossus*) and turbot (*Scophthalmus maximus*). J Vet Pharmacol Ther. (1999) 22:122–6. 10.1046/j.1365-2885.1999.00191.x 10372596

[26] Kverme KO Haugland GT Hannisdal R Kallekleiv M Colquhoun DJ Lunestad BT Pharmacokinetics of florfenicol in lumpfish (*Cyclopterus lumpus* L.) after a single oral administration. Aquaculture. (2019) 512:734279 10.1016/j.aquaculture.2019.734279

[27] Yamaoka K Nakagawa T Uno T. Application of Akaike‘s information criterion (AIC) in the evaluation of linear pharmacokinetic equations. J Pharmacokinet Biopharm. (1978) 6:165–75. 10.1007/BF01117450 671222

[28] Glass GV Peckham PD Sanders JR Consequences of failure to meet assumptions underlying the fixed effects analyses of variance and covariance. Rev Educ Res. (1972) 42:237–88. 10.3102/00346543042003237

[29] Stubbings G Bigwood T The development and validation of a multiclass liquid chromatography tandem mass spectrometry (LC-MS/MS) precedure for the determination of veterinary drug residues in animal tissue using a QuEChERS (Quick, Easy, Cheap, Effective, Rugged and Safe) approach. Anal Chim Acta. (2009) 637:68–78. 10.1016/j.aca.2009.01.029 19286014

[30] Ishida N Tissue levels of oxolinic acid after oral or intravascular administration to freshwater and seawater rainbow trout. Aquaculture. (1992) 102:9–15. 10.1016/0044-8486(92)90284-R

[31] Rogstad A Ellingen OF Syvertsen C Pharmacokinetics and bioavailability of flumequine and oxolinic acid after various routes of administration to Atlantic salmon in seawater. Aquaculture. (1993) 110:207–20. 10.1016/0044-8486(93)90369-A

[32] Rigos G Tyrpenou AE Nengas I Alexis M Troisi G The kinetic profile of oxolinic acid in sharpsnout sea bream, *Diplodus puntazzo* (Cetti 1777). Aquacult Res. (2004) 35:1299–304. 10.1111/j.1365-2109.2004.01127.x

[33] Hansen MK Horsberg TE. Single-dose pharmacokinetics of flumequine in cod (*Gadus morhua*) and goldsinny wrasse (*Ctenolabrus rupestris*). J Vet Pharmacol Ther. (2000) 23:163–8. 10.1046/j.1365-2885.2000.00259.x 11110104

[34] Elema MO Hoff KA Kristensen HG Bioavailability of flumequine after oral administration to Atlantic salmon (*Salmo salar* L). Aquaculture. (1995) 136:209–19. 10.1016/0044-8486(95)01049-1

[35] Elema MO Hoff KA Kristensen HG Multiple-dose pharmacokinetic study of flumequine in Atlandic salmon (*Salmo salar* L.). Aquaculture. (1994) 128:1–11. 10.1016/0044-8486(94)90096-5

[36] Shojaee Aliabadi F Lees P Antibiotic treatment for animals: effect on bacterial population and dosage regimen optimisation. Int J Antimicrob Agents. (2000) 14:307–13. 10.1016/S0924-8579(00)00142-4 10794952

[37] Barnes AC Lewin CS Hastings TS Amyes SG. *in vitro* susceptibility of the fish pathogen *Aeromonas salmonicida* to flumequine. Antimicrob Agents Chemother. (1991) 35:2634–5. 10.1128/AAC.35.12.2634 1810198 PMC245444

[38] Wright DH Brown GH Peterson ML Rotschafer JC Application of quinolones pharmacodynamics. Int J Antimicrob Agents. (2000) 46:669–83. 10.1093/jac/46.5.669 11062185

[39] Björklund HV Bylund G. Comparative pharmacokinetics and bioavailability of oxolinic acid and oxytetracycline in rainbow trout (*Oncorhynchus mykiss*). Xenobiotica. (1991) 21:1511–20. 10.3109/00498259109044401 1763525

[40] Plakas SM El Said KR Musser SM Pharmakokinetics, tissue distribution, and metabolism of flumequine in channel catfish (*Ictalurus punctatus*). Aquaculture. (2000) 187:1–14. 10.1016/S0044-8486(00)00303-3

