# Supplementary figures and images for: Pharmacokinetic Data Show That Oxolinic Acid and Flumequine Are Absorbed and Excreted Rapidly From Plasma and Tissues of Lumpfish
Source: Front Vet Sci. 2019 Nov 12;6:394. doi: 10.3389/fvets.2019.00394 (PMC6861182; doi:10.3389/fvets.2019.00394)

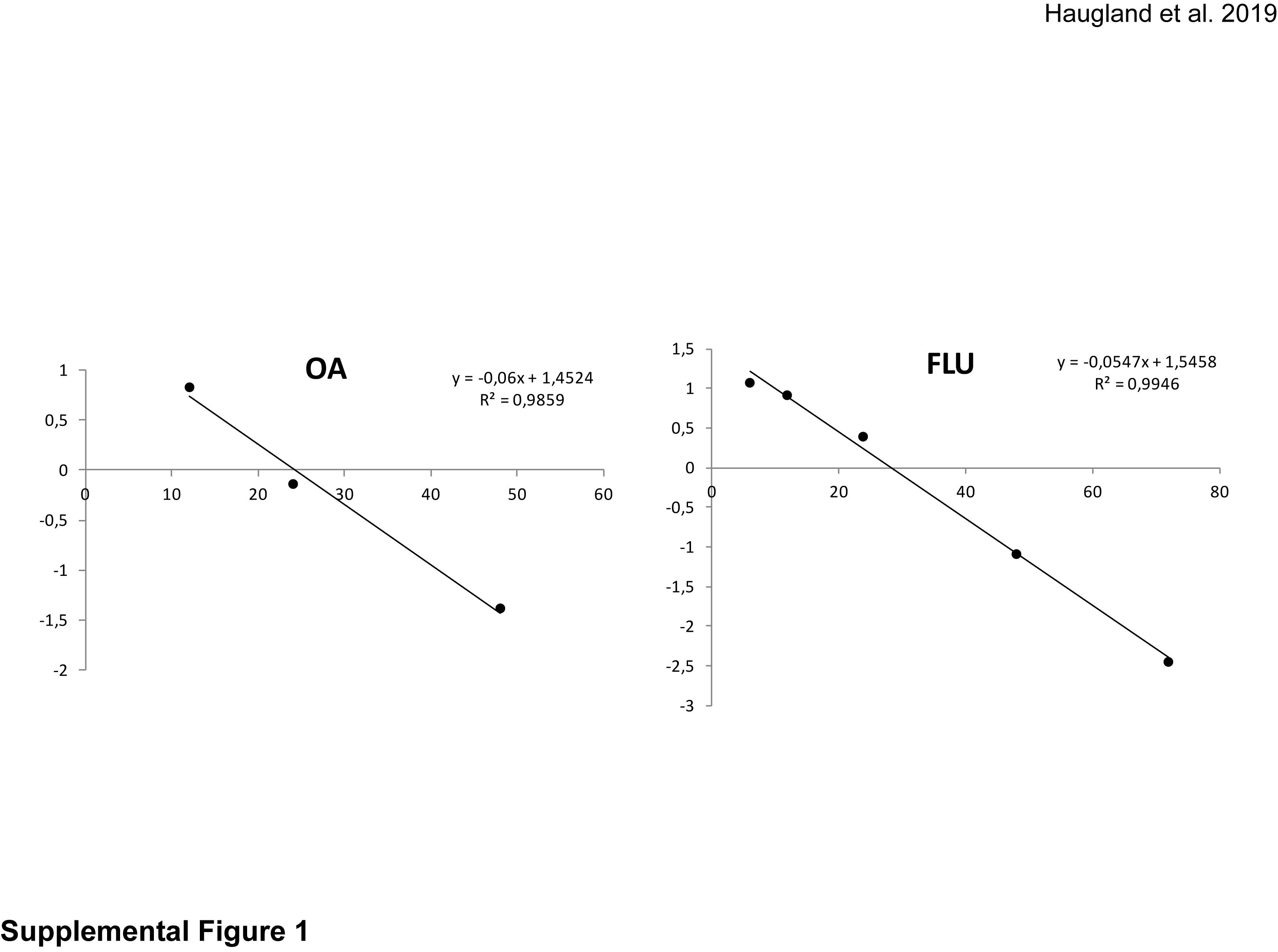

Supplement: Supplementary file 1 [file Image_1.TIF]
